# Genetic polymorphisms in serine protease inhibitor Kazal-type 5 and risk of atopic dermatitis: A meta-analysis

**DOI:** 10.1097/MD.0000000000021256

**Published:** 2020-07-10

**Authors:** Yunling Li, Yin Li, Wei Li, Xiaoxuan Guo, Sha Zhou, Huiwen Zheng

**Affiliations:** Department of Dermatology, Children's Hospital, Zhejiang University School of Medicine, National Clinical Research Center for Child Health, Hangzhou 310052, Zhejiang Province, China.

**Keywords:** atopic dermatitis, meta-analysis, polymorphism, risk factor, *SPINK5*

## Abstract

**Background:**

This study aimed to investigate the role of serine protease inhibitor Kazal-type 5 (*SPINK5*) polymorphisms (Asn368Ser, Asp386Asn and Glu420Lys) and the risk of atopic dermatitis (AD).

**Methods:**

Studies associated with *SPINK5* mutations and AD risk were searched from three databases, including PubMed, Embase, and Cochrane library, with a retrieval deadline of April 22, 2019. An odds ratio (OR) with a 95% confidence interval (95% CI) was chosen as the effect size. Egger's linear regression test was enrolled to assess the level of publication bias.

**Results:**

Overall, 6 studies met the inclusion criteria for meta-analysis. Significantly statistical differences were calculated between patients with AD and healthy individuals on Asn368Ser polymorphism in the allele model (G vs A: OR = 1.2643, 95% CI = 1.0666–1.4987, *P* = .0069), co-dominant model (GG vs AA: OR = 1.6609, 95% CI = 1.1736–2.3505, *P* = .0042; GA vs AA: OR = 1.5448, 95% CI = 1.1263–2.1189, *P* = .0070), and dominant model (GG+GA vs AA: OR = 1.5700, 95% CI = 1.1656–2.1146, *P* *=* .0030). However, no statistically significant difference was found in the recessive model for Asn368Ser and other genetic models for Asp386Asn and Glu420Lys (all *P* > .05). No significant publication bias was found.

**Conclusion:**

The *SPINK5* Asn368Ser polymorphism may be a risk factor for AD.

**Key points**

- The role of *SPINK5* in atopic dermatitis (AD) risk was meta-analyzed.
- *SPINK5* Asn368Ser was significantly associated with AD risk.
- Polymorphisms of Asp386Asn and Glu420Lys were not associated with AD risk.

## 1 Introduction

Atopic dermatitis (AD), also known as atopic eczema, is considered a chronic inflammatory skin disease. It results in dry, itchy, swollen, and red skin. As of 2018, the point prevalence of adult AD in the overall/treated populations was 4.9%/3.9% in the United States, 3.5%/2.6% in Canada, 4.4%/3.5% in the EU, and 2.1%/1.5% in Japan,^[1]^ and the prevalence of AD continues to increase in developing countries.^[2]^ Traditionally, AD is often associated with abnormalities in the skin barrier and immune system dysfunction, accompanied by high microbial colonization and a higher susceptibility to skin infection.^[3,4]^ However, the pathogenesis of AD is not fully understood.

Serine protease inhibitor Kazal-type 5 (*SPINK5*) is a member of the gene family serine protease inhibitor Kazal-type cluster located on chromosome 5q32, which encode inhibitors of serine proteases. The encoded proteins are mainly distributed in the vaginal epithelium, thymus, vestibular gland, oral mucosa, tonsils, and parathyroid glands, which are mainly involved in the hydrolysis of human growth hormone and skin desquamation.^[5]^ Various mutations in *SPINK5* have been identified in patients with AD, and results were widely variable. For example, data from the study by Nishio et al. showed that five missense mutations, such as Asn368Ser, Asp386Asn, and Glu420Lys were associated with AD.^[6]^ However, Jongepier and his colleagues demonstrated that *SPINK5* was not associated with atopic phenotypes in individuals ascertained by a proband with asthma.^[7]^ Thus, the association of AD with *SPINK5* polymorphisms remains unclear, and results are not conclusive. Therefore, a meta-analysis would be needed to evaluate the role of *SPINK5* polymorphisms and the risk of AD.

In this meta-analysis, previous studies associated with *SPINK5* mutations (Asn368Ser, Asp386Asn and Glu420Lys) and AD risk were searched. An odds ratio (OR) with a 95% confidence interval (95% CI) was chosen as the effect size to explore the potential association between *SPINK5* polymorphisms and AD risk.

## 2 Methods

The meta-analysis was performed following the guidelines provided by the Preferred Reporting Items for Systematic Reviews and Meta-Analysis Protocols (PRISMA-P). Ethical approval was not necessary since this is a meta-analysis and no patients or animals involved.

### 2.1 Search strategy

Electronic English works of literature were searched from databases, including PubMed (http://www.ncbi.nlm.nih.gov/pubmed/), Embase (http://www.embase.com), and Cochrane library (http://www.cochranelibrary.com/) with a retrieval deadline of April 22, 2019, based on the predefined search strategy. The keywords and search terms used for all searches were “atopic dermatitis” or “AD” AND “SPINK5” OR “serine protease inhibitor kazal type 5” or “serine protease inhibitor Kazal-type 5” and “SNP” or “Single Nucleotide Polymorphism” or “polymorphism” or “genetic” or “variant”. Finally, in order to enroll more studies, articles of paper literature, and citations were manually screened.

### 2.2 Inclusion and exclusion criteria

The present meta-analysis would include the following studies: (1) the research design was a case-control study; (2) the participants in the case group were patients diagnosed with AD, and the participants in the control group were healthy people or hospitalized patients diagnosed without AD; (3) the association between *SPINK5* polymorphism and AD was investigated; (4) data associated with the genotype and allele frequency of *SPINK5* polymorphism (Asn368Ser, Asp386Asn and Glu420Lys) were provided.

Types of literature would be excluded if they were (1) studies with incomplete data, and statistical analysis could not be performed; (2) reviews, letters, and/or comments. For duplicated publications, only the study with the most complete data, most updated data, or higher Newcastle-Ottawa Scale (NOS) score could be included.

#### 2.2.1 Data extraction and quality evaluation

The authors independently evaluated all relevant articles and extracted relevant data: the first author name, publication year, study region, diagnostic criteria of AD, the detection method of genotype, the same size in the case group and control group, gender, age, and outcomes in each group. Any discrepancies would be resolved by discussion. The genetic polymorphisms mainly included Asn368Ser, Asp386Asn, and Glu420Lys.

The NOS was used to assess the quality of enrolled studies,^[8]^ which was recommended by the Agency for Healthcare Research and Quality (AHRQ) for quality assessment of each case-control study. For example, NOS scores of 0-3, 4-6, and 7-9 represented low, moderate, and high-quality studies, respectively.

### 2.3 Statistical analysis

Data analyses in this study were performed using the R software package 3.12. We initially assessed whether the genotype distribution in the control group was in accordance with Hardy Weinberg Equilibrium (HWE) by using the Chi-square test.^[9]^ ORs with their 95% CI^[10]^ of the allele model, co-dominant model, recessive model, and dominant model were calculated in order to assess the relationship between *SPINK5* polymorphisms and AD. The heterogeneity was assessed by Dixon's *Q*-test^[11]^ and *I*^2^ test. We defined that significant heterogeneity occurred if *P* < .05 or *I*^2^ > 50%, and then data would be pooled by the random effects model.^[12]^ If *P* value >.05 or *I*^2^ < 50%, data would be pooled by the fixed effect model.^[13]^ The publication bias was evaluated using the Egger's linear regression test^[14]^ with *P* > .05, indicating no publication bias. Sensitivity analysis was performed by eliminating one study at each defined interval. The results are stable if the outcomes did not change.

## 3 Results

### 3.1 Study selection

**Figure 1:**
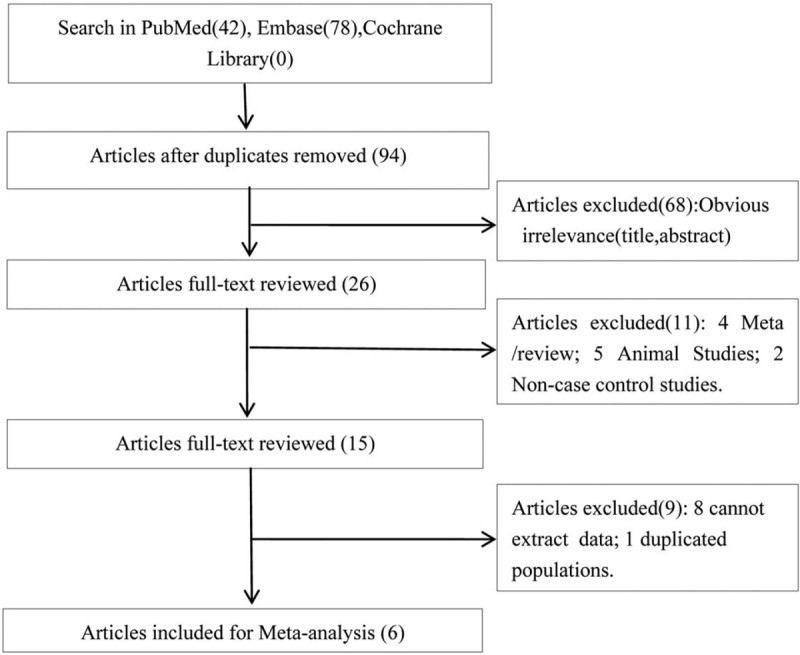
Literature search and study selection.

Figure 1 shows the process of study selection in detail. Initially, a total of 120 potentially relevant papers were retrieved (PubMed: n = 42; Embase: n = 78; Cochrane Library: n = 0). Twenty-six duplicate articles were excluded by screening the titles. Next, 68 irrelevant articles were excluded after reading the title and abstract. For the remaining 26 publications, 20 (5 animal studies, 2 non-case and control studies, 4 meta-analyses/reviews, 1 duplicated population study, and 8 studies in which genotype data could not be obtained) articles were excluded by reviewing the full text. Finally, 6 articles met the inclusion criteria^[7,15–19]^ and were included in the meta-analysis.

### 3.2 Study characteristics and quality assessment

The included study characteristics are collected in Table 1. All 6 included articles are case-control studies and good quality studies with a NOS score ranging from 6 to 9. These studies were conducted in China, Japan, the United States, and Germany, and were published between 2003 and 2018. A total of 1968 participants were enrolled in this meta-analysis, including 914 patients with AD in the case group and 1054 participants in the control group. The diagnostic criteria of AD were mainly Hanifin and Rajka Criteria.^[20]^ The genotype detection methods were polymerase chain reaction (PCR)-restriction fragment length polymorphism analysis (PCR-RFLP) and/or PCR amplification. Most articles did not report on the gender ration. Table 2 shows the *SPINK5* gene polymorphisms [A1103G (Asn368Ser), G1156A (Asp386Asn), and G1258A (Glu420Lys)] of each included study. It is worth noting that the genotype distribution in the control group of one study ^[19]^ deviated from HWE.

**Table 1:**
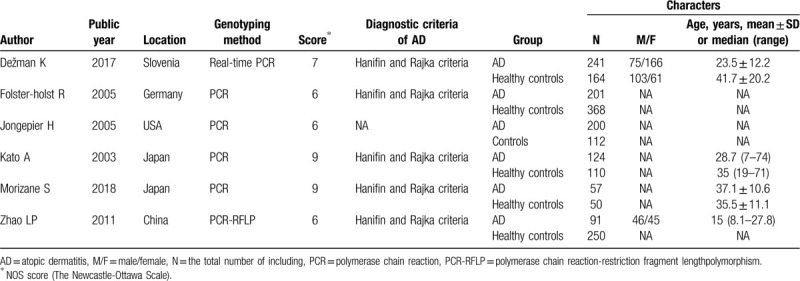
Characteristics of included studies.

**Table 2:**
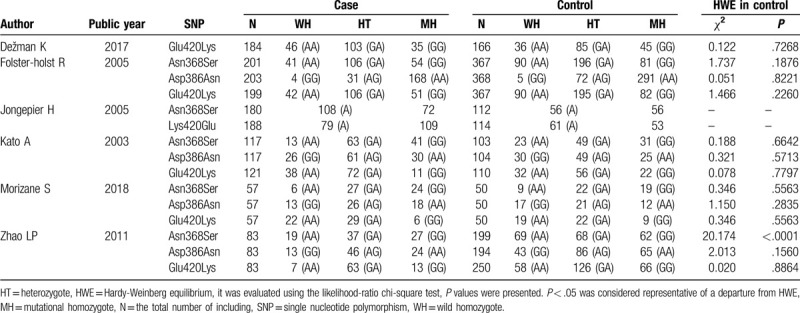
Gene distribution of included studies.

### 3.3 Meta-analysis for the association between SPINK5 polymorphism and AD

The associations between AD and the genetic polymorphisms of different genetic models for *SPINK5* were evaluated in this study. The allele model (Asn368Ser: G vs A; Asp386Asn: A vs G; Glu420Lys: G vs A), co-dominant model (Asn368Ser: GG vs AA, GA vs AA; Asp386Asn: AA vs GG, AG vs GG; Glu420Lys: GA vs AA, GG vs AA), recessive model (Asn368Ser: GG vs AA+GA; Asp386Asn: AA vs GG+AG; Glu420Lys: GG vs AA+GA), and dominant model (Asn368Ser: GG+GA vs AA; Asp386Asn: AA+AG vs GG; Glu420Lys: GG+GA vs AA) for Asn368Ser, Asp386Asn, and Glu420Lys were evaluated.

The heterogeneity test results showed significant heterogeneity among the genetic models of GA vs AA, GG vs AA, GG vs AA+GA, and GG+GA vs AA for Glu420Lys (*P* < .05, *I*^2^ > 50%, Table 3). Therefore, data among individual studies were pooled using the random effects model. The fixed effects model was applied to other genetic models (Table 3).

**Table 3:**
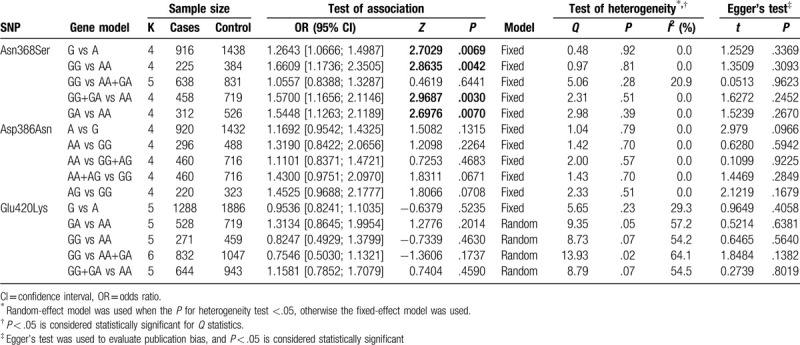
Meta-analysis of the association between genetic polymorphism of SPINK5 and AD.

**Figure 2:**
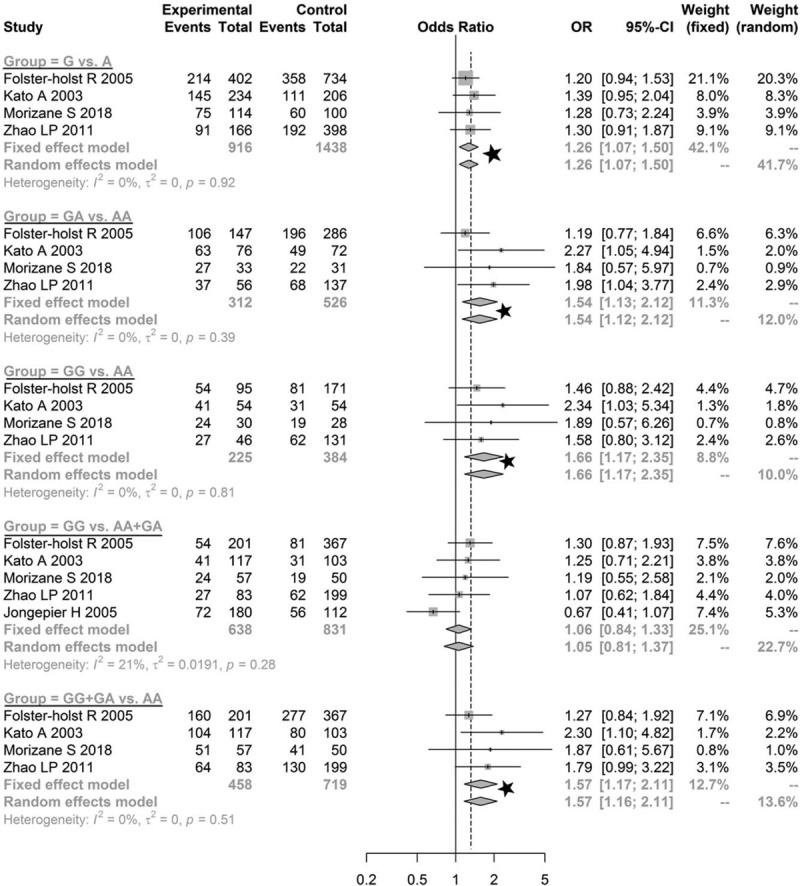
Meta-analysis of the association between genetic models of Asn368Ser and atopic dermatitis. The significant results were marked with “☆”.

**Figure 3:**
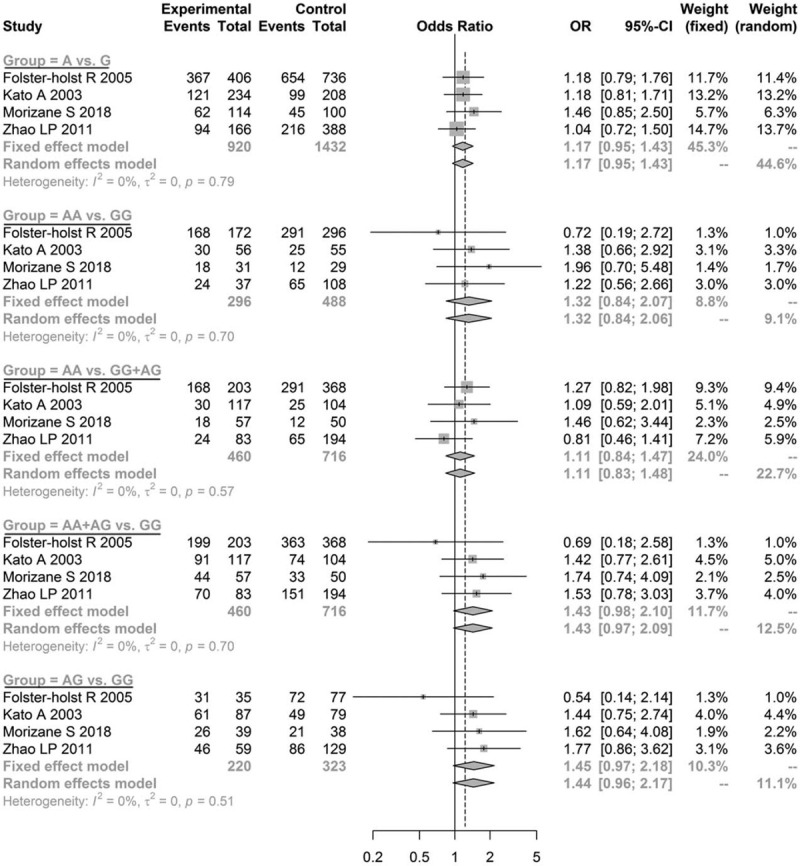
Meta-analysis of the association between genetic models of Asp386Asn and atopic dermatitis.

**Figure 4:**
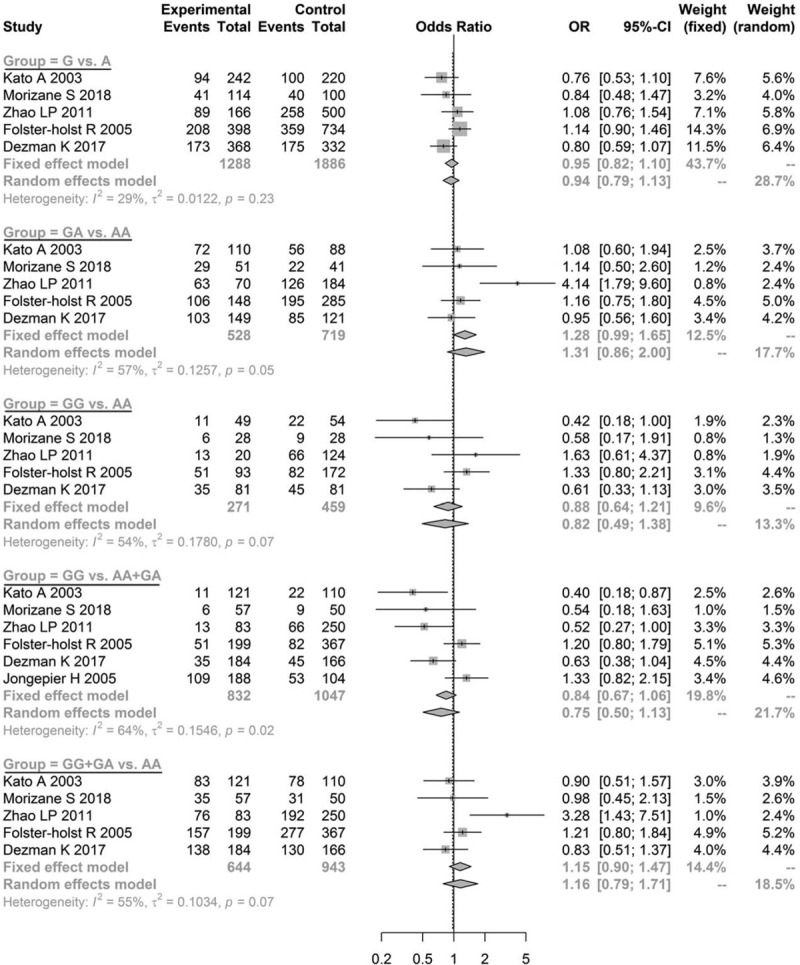
Meta-analysis of the association between genetic models of Glu420Lys and atopic dermatitis.

The pooled estimates for Asn368Ser of the allele model (G vs A: OR = 1.2643, 95% CI = 1.0666–1.4987, *P* = .0069), co-dominant model (GG vs AA: OR = 1.6609, 95% CI = 1.1736–2.3505, *P* = .0042; GA vs AA: OR = 1.5448, 95% CI = 1.1263–2.1189, *P* = .0070), and dominant model (GG+GA vs AA: OR = 1.5700, 95% CI = 1.1656–2.1146, *P* *=* .0030) indicated significantly statistical differences, while the pooled estimates of the recessive model (GG vs AA+GA: OR = 1.0557, 95% CI = 0.8388–1.3287, *P* *=* .6441) were not significantly different (Fig. 2). Furthermore, since the OR value and its 95% CI were both greater than 1, the mutation of Asn368Ser in *SPINK5* was determined to be a risk factor for AD. No statistically significant difference was found in the other genetic models for Asp386Asn (Fig. 3) and Glu420Lys (Fig. 4, all *P* > .05). These results demonstrated that the genetic polymorphism of Asn368Ser of *SPINK5* was significantly related to AD morbidity and is a risk factor for AD.

### 3.4 Sensitivity analysis and publication bias

In the sensitivity analysis, the meta-analysis results of the genetic model GA vs AA of Asn368Ser, AA+AG vs GG of Asp386Asn, and AG vs GG of Asp386Asn were changed, while the other results were not changed. These results indicated the relative stability and reliability of the results. Additionally, Egger's test (*P* > .05) showed that publication bias among studies was not significant (Table 3).

## 4 Discussion

In our study, 6 articles were included in the meta-analysis. The statistically significant difference between patients with AD and healthy participants was calculated for the Asn368Ser polymorphism of the allele model, co-dominant model, and dominant model. However, no significant difference was found in the recessive model for Asn368Ser and other genetic models for Asp386Asn and Glu420Lys. Thus, our data suggest the *SPINK5* Asn368Ser polymorphism may be a risk factor for AD.

It is well known that the skin acts as an essential barrier against pathogens and exogenous agents. Moreover, skin barrier dysfunctions are one of the major factors involved in AD development.^[2]^ The gene *SPINK5* is located on chromosome 5q31-32, which encodes the skin barrier protein lympho-epithelial Kazal-type-related inhibitor (also known as serine protease inhibitor Kazal-type 5).^[21,22]^*SPINK5* in the epidermis is primarily expressed in the stratum granulosum, where it functions as a protease. Thus, it is important in the cornification of epithelial differentiation and exfoliation.^[23,24]^ Previous evidence demonstrated that *SPINK5* could prevent an influx of pathogens based on the formation of the cornified cell envelope.^[5]^ Evidence from a study by Mocsai et al. showed that skin barrier functions were related to total immunoglobulin E (IgE) levels.^[25]^ Furthermore, Tanei and his colleagues showed that allergic inflammation, mediated by the level of IgE, was crucial in the pathobiology of AD.^[26]^ Recently, Hubiche et al. found an association between *SPINK5* E420K polymorphisms and high IgE serum levels.^[27]^ Moreover, several extensive studies support the role of SPINK5 in the development of AD.^[16,18]^ Thus, the mechanism and the potential role of *SPINK5* should be fully elucidated in future studies.

Notably, significant heterogeneity was calculated among data evaluating *SPINK5* Glu420Lys polymorphism genotypes, including GA vs AA, GG vs AA, GG vs AA+GA, and GG+GA vs AA. Recently, it was determined that AD in several ethnic groups displayed variant mutation spots and rates between populations.^[28]^ Moreover, skin barrier dysfunctions introduced by virulence factors could also induce allergic inflammation via innate and adaptive immunity.^[29]^ Thus, limited background information of enrolled patients from a different ethnicity might be possible sources of heterogeneity. Although no significant difference was found in the recessive model for Asn368Ser and other genetic models for Asp386Asn and Glu420Lys in the meta-analysis, further clinical data would also be needed to verify the conclusion.

Furthermore, limitations of this meta-analysis should be noted. Firstly, the enrolled number of patients was small, and subgroup analysis could not be performed. Secondly, the genotyping method was different among enrolled studies, and the sensitivity ability of each method varied, which might lead to false-negatives. Third, though we found the association of *SPINK5* Asn368Ser polymorphism and risk of AD, whether this SNP could influence gene expression of *SPINK5* should be further investigated. Therefore, a study with higher NOS scores and larger sample size would be needed.

In conclusion, our study supports the role of *SPINK5* Asn368Ser polymorphism as one of the risk factors for patients with AD. Future studies fully elucidating the pathogenic mechanisms involved in the disease are needed.

## Author contributions

YLL and HWZ designed the study. YLL received the fund and was a major contributor in drafting the manuscript. HWZ revised the manuscript. YL, WL and XXG searched the references, reviewed the references and extracted the data. SZ performed quality evaluation and statistical analysis. All authors reviewed and approved the final version of the manuscript.
